# La gestión de la demanda de las pruebas: un reto en el nuevo modelo de medicina de laboratorio

**DOI:** 10.1515/almed-2023-0146

**Published:** 2024-04-26

**Authors:** María Salinas, Ruth Torreblanca, Eduardo Sanchez, Álvaro Blasco, Emilio Flores, Maite López-Garrigós

**Affiliations:** 16805 Servicio de Análisis Clínicos, Hospital Universitario San Juan de Alicante, San Juan de Alicante, España; Departamento de Medicina Clínica, Universidad Miguel Hernández, San Juan de Alicante, España; Departamento de Bioquímica y Biología Molecular, Universidad Miguel Hernández, Elche, España; CIBER en Epidemiología y Salud Pública (CIBERESP), Madrid, España

**Keywords:** adecuación, gestión de la demanda, gestión del resultado, Medicina de Laboratorio

## Abstract

**Introducción:**

En las últimas décadas está evolucionando el posicionamiento de la Medicina de Laboratorio en el proceso médico asistencial de atención al paciente y también el modelo de laboratorio; de un modelo tradicional, con solo intervención, a un modelo líder, que además de intervenir condiciona la decisión clínica. La mejora en tecnología y automatización ha permitido también al profesional de laboratorio centrarse en la primera y última fase del ciclo de laboratorio, la solicitud de pruebas, y la acción tras su resultado, las etapas con más errores, y donde principalmente se debe actuar para conseguir una mejora en la calidad asistencial del paciente.

**Contenido:**

Se muestra el diseño y la implantación de intervenciones de gestión de la demanda de pruebas de laboratorio, logrando el diagnóstico de la enfermedad oculta, y mejorando la adherencia a las guías clínicas y la seguridad del paciente.

**Resumen:**

Se expondrán los puntos clave en el proceso de gestión de la demanda, tanto de exceso como de defecto.

**Perspectivas:**

El objetivo de la revisión es lograr que el profesional del laboratorio se involucre en el diseño e implantación de intervenciones de gestión de la demanda y en la creación de ese nuevo modelo de Laboratorio Líder.

## Introducción

El proceso de laboratorio clínico es un proceso multifásico que comienza cuando el médico solicita las pruebas de laboratorio y finaliza con la toma de decisiones acorde a sus resultados [1]. La Medicina de Laboratorio (ML) es la especialidad médica que interviene más frecuentemente [2] en el proceso de atención al paciente, siendo clave la seguridad del laboratorio para lograr la seguridad del paciente [3].

Debido a las mejoras tecnológicas, en la actualidad la etapa analítica es, de todo el ciclo de laboratorio, donde se refieren menos errores, siendo las pre- y post-analíticas donde se describen mayor número de errores de laboratorio [4]. Especialmente, donde se ha referido que se cometen más errores y en las etapas en las que principalmente se debe actuar para mejorar la calidad asistencial del paciente, son la primera, la etapa pre-preanalítica, y la última, la etapa post-postanalítica [5, 6].

Además, en estas últimas décadas han ocurrido una serie de cambios que han contribuido también a un cambio en el posicionamiento de la ML.

En primer lugar, la mayor intervención en el proceso de atención al paciente. Hace 40 años solo intervenía en el 10–15 % de las decisiones clínicas [7] y ya se ha referido actualmente una participación de la ML en la mayoría de las decisiones clínicas [2].

En segundo lugar, en la actualidad existen determinadas pruebas que, a determinados puntos de corte, constituyen criterios para la toma de decisiones en el diagnóstico, el tratamiento, el pronóstico o la utilización o no de diferentes herramientas diagnósticas, como podría ser el caso de realización de una biopsia ante un determinado resultado de antígeno prostático específico. Era impensable hace 30 años, que una prueba de laboratorio fuera la que decidiera si el paciente presentaba o no un infarto de miocardio como ocurre con la troponina en la actualidad [8]. Igualmente ocurre con el diagnóstico de daño renal, son el filtrado glomerular y la concentración de albúmina en orina los factores que intervienen en el diagnóstico [9]. Es clave seleccionar pruebas en base a la evidencia y a recomendaciones en dichas guías clínicas [10].

El tercer y último factor que ha propiciado el cambio en el posicionamiento de la ML es la aparición de las soluciones digitales, los sistemas de ayuda a la decisión clínica (CDS) y la inteligencia artificial [11]. Estos sistemas están siendo relativamente fáciles de incorporar a la labor diaria de los laboratorios clínicos, dada la costumbre de estos servicios en el manejo de sistemas de información, aunque el hecho de usar un sistema de información de laboratorio (SIL) no se asocia con la facilidad de uso e implantación de herramientas de inteligencia artificial, sí puede predisponer a una mejor adaptación a estos sistemas.

Este cambio en el posicionamiento de la ML implica también un cambio paralelo en el modelo de laboratorio, y también en el papel del profesional del laboratorio clínico. De hecho, es el profesional del laboratorio clínico el que tiene el conocimiento sobre la prueba de laboratorio; y además un conocimiento que no es suficientemente impartido en las facultades de Medicina. Parece que la formación en las ciencias del laboratorio clínico, que no era suficientemente hace más de 100 años [12], sigue sin serlo 100 años después, como se demostró en un estudio respecto al grado del conocimiento del médico de la variabilidad biológica y analítica, tan importante para la correcta interpretación de las pruebas de laboratorio [13]. Es por todo ello que el profesional de laboratorio, en este nuevo rol de liderar el buen uso de las pruebas, tiene que posicionarse como experto, y proporcionar conocimiento y no solo datos, debido a la relevancia que tienen los resultados en la mayoría de las decisiones clínicas [14]. También, es responsabilidad del profesional de laboratorio evitar una solicitud inadecuada y los efectos adversos que de ella puedan generarse [15]. Otras competencias que debe integrar un buen profesional de laboratorio clínico incluyen valores como la imaginación creativa, el liderazgo y la comunicación con el clínico [16]. La comunicación es un valor fundamental ante cualquier intervención liderada desde el laboratorio [17]. Además del cambio descrito en el papel del profesional del laboratorio, el cambio en el posicionamiento de la ML también genera una modificación en el modelo de laboratorio [18]. De un laboratorio tradicional, o tecnológico, que solo interviene en la decisión clínica, a un Laboratorio Líder que adicionalmente “hace” la decisión clínica. Lo que principalmente diferencia al Laboratorio Líder del tradicional, o incluso del modelo intermedio (el laboratorio tecnológico) son dos hechos: (1) el diseño y establecimiento de Intervenciones de Gestión de Resultado (IGR), se confirma que el resultado de la prueba ha sido comunicado, recibido y que se ha tomado la acción adecuada, y; (2) el diseño y establecimiento de Intervenciones de Gestión de la Demanda (IGD) que corrigen el defecto o el exceso en la solicitud de las pruebas de laboratorio. En el modelo tradicional no existen esos dos tipos de intervenciones, y en el tecnológico como mucho solo se implementan las IGD que corrigen el exceso de solicitud. Y la diferencia entre estos dos tipos de actuaciones es enorme. Por ejemplo, cuando se corrige el exceso de la demanda de hemoglobina glicada (HbA_1c_), solo nos ahorramos el gasto de reactivo. Cuando se corrige el defecto, midiendo esta prueba de forma automática en personas, incluso jóvenes, que presentan alteraciones en el perfil lipídico, tal como recomiendan las guías [19], es cuando realmente se beneficia no solo al paciente, sino también a la sociedad, por el diagnóstico temprano de diabetes [20]. Así, en este Laboratorio Líder, se consigue el diagnóstico de la enfermedad oculta, y ese diagnóstico temprano mejorará considerablemente el pronóstico de la enfermedad.

Además, en el Laboratorio Líder se monitoriza de forma continua en el tiempo cómo desde el laboratorio se mejora la calidad asistencial del paciente mediante indicadores de resultado [21]. De forma adicional a los indicadores clásicos, que podrían denominarse intermedios, como son número de pruebas, pruebas por paciente, incidencias en las muestras, tiempo de respuesta, etc., se contabilizan estos indicadores de resultado de los que se pueden poner como ejemplo el número de pacientes que se ha identificado con una enfermedad oculta o el aumento en la prescripción de vitamina B12 intramuscular en atención primaria (AP) ante la nueva identificación de pacientes con déficit grave de vitamina B12 [18]. Por tanto, debe quedar patente que nuestra misión, la misión de la ML, no es el procesamiento de pruebas, sino la prevención, el diagnóstico, la monitorización y el tratamiento de la enfermedad. Y estos indicadores de resultado o finales, es lo que miden. Así desde este Laboratorio Líder, y con esos valores del profesional del laboratorio clínico; conocimiento, comunicación, liderazgo e imaginación creativa, se va logrando alcanzar la misión, que no es otra que, desde la ML lograr el máximo beneficio para el ciudadano, el paciente y la sociedad.

Esta revisión no pretende ser un tratado académico respecto a lo que es la gestión de la demanda, ni un repaso exhaustivo de todas las intervenciones publicadas. El objetivo es lograr que el profesional del laboratorio se involucre en el diseño e implantación de IGD. Este objetivo se intentará alcanzar, en primer lugar, exponiendo los puntos que son clave en este proceso de diseño e implantación de intervenciones, y posteriormente enumerando y explicando IGD de corrección tanto del exceso como del defecto de demanda, en las que los autores tienen una experiencia consolidada. Todas estas IGD están muy bien detalladas en publicaciones de alto impacto, y por ello son muy fáciles de replicar. También es objetivo de esta revisión que el profesional de laboratorio se involucre en la creación de ese nuevo modelo, el Laboratorio Líder, tan beneficioso no solo para el ciudadano, el paciente y la sociedad, sino también para la supervivencia del laboratorio clínico, un proceso clave y no de apoyo [22] en este proceso global multifásico de atención al paciente.

## Efectos adversos y necesidad de corregir la inadecuación en la solicitud de pruebas de laboratorio

Fryer A et al., en una excelente revisión acerca del tema de la adecuación de la solicitud de pruebas de laboratorio [23], categorizaron, definieron y cuantificaron la solicitud inapropiada: “Una solicitud (lo que implica que es pedida por un solicitante) que se hace al margen de algún tipo de recomendación acordada (incluyendo las solicitadas demasiado tarde)”. Una solicitud inapropiada es aquella que generalmente no debe ser procesada, ya que se solicita en el paciente equivocado, en el momento equivocado, para el proceso inadecuado o la prueba equivocada [24].

La corrección de la solicitud inapropiada de pruebas de laboratorio es un gran reto para el profesional del laboratorio, debido a sus elevados y conocidos efectos adversos.

El defecto de la solicitud genera como efecto adverso la no realización de nuestra misión, de prevención, diagnóstico, monitorización y/o tratamiento de la enfermedad. De hecho, se puede estar perdiendo un diagnóstico por no solicitar una prueba de laboratorio acorde a la situación del paciente, o dejando de monitorizar a un enfermo crónico acorde a las guías por no estar solicitadas las pruebas recomendadas en dichas guías.

Por otro lado, el exceso en la solicitud de pruebas también genera efectos adversos, no solo los costes de la prueba en sí. Las pruebas de laboratorio pertenecen al grupo denominado “*little ticket tests*” [25], aquella prueba que es “barata” individualmente pero que como es altamente solicitada genera un alto coste. Además del aumento de gasto, la inadecuación por exceso produce los efectos adversos de los resultados falsos positivos generados, al ser solicitadas las pruebas en poblaciones de baja prevalencia de la enfermedad [15]. Los efectos colaterales del síndrome de Ulises [26] y del síndrome del Enfermo Imaginario [27] son consultas médicas innecesarias y pruebas diagnósticas adicionales, que generalmente generan más costes que la prueba solicitada en exceso. Y el tercer efecto adverso del exceso de demanda es la contribución al exceso de pruebas de laboratorio. El laboratorio se mercantiliza, se convierte en una máquina expendedora de datos, y no en un proveedor de conocimiento. El profesional de laboratorio no dispone de tiempo para informar conocimiento, solo datos [14], y el resultado útil para la decisión clínica adecuada puede quedar oculto por los datos no útiles o comparsa, lo que implicaba un mayor riesgo de que el clínico no ejerza la acción adecuada tras la recepción del informe de laboratorio.

**Figura 1:**
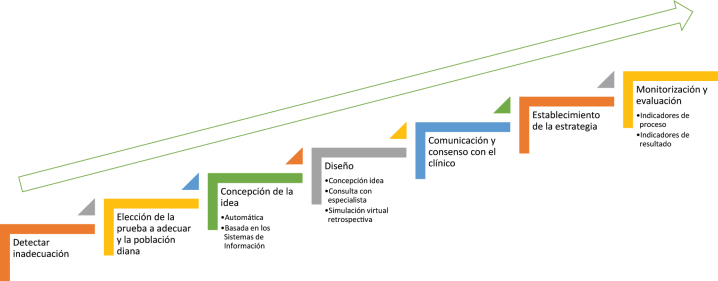
Fases en el diseño y establecimiento de Intervenciones de gestión de la demanda (IGD).

Todo lo expuesto indica que, dada la alta demanda de pruebas y la alta intervención de la ML en el proceso de atención al paciente, son muy importantes las IGD para las pruebas de laboratorio, y su diseño e implementación, que implica un conocimiento y seguimiento de todas sus fases, desde la detección de las pruebas inadecuadamente solicitadas, primer paso, hasta la monitorización continua mediante indicadores de resultado, como último paso [28] (Figura 1).

## Fases en el diseño y establecimiento de intervenciones de gestión de la demanda

### Identificación de la prueba inadecuadamente solicitada

**Figura 2:**
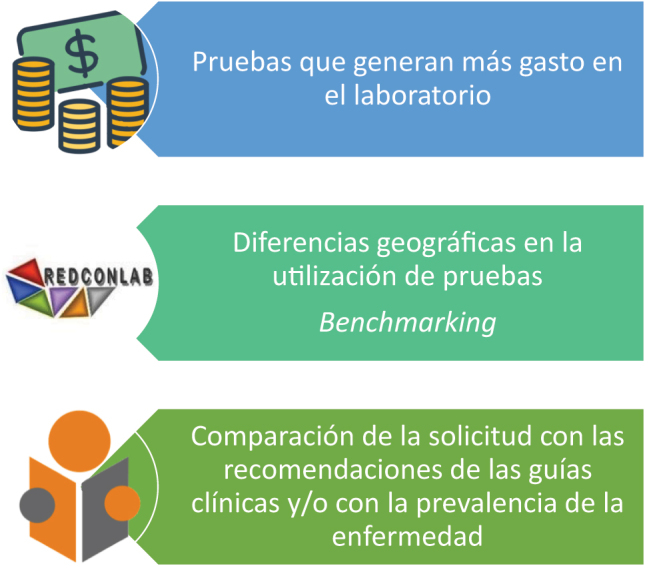
¿Cómo detectar inadecuación?

El primer paso, la detección de la prueba inadecuadamente solicitada, tradicionalmente se ha realizado mediante la revisión de historias clínicas [29] para tratar de averiguar si la solicitud de la prueba era acorde o no a la situación clínica del paciente. La revisión de historias clínicas son el fundamento de estudios retrospectivos, largos en el tiempo y muy costosos. Sin embargo, últimamente se han formulado maneras indirectas para la detección de la inadecuación (Figura 2).

Al menos en un entorno de sistemas sanitarios de gestión pública, uno de los métodos indirectos sería mediante el estudio de las pruebas de laboratorio que suponen al laboratorio un mayor gasto. Aunque en principio esto no implica un exceso de solicitud de la prueba, sí que es un buen comienzo para, a partir de ahí, estudiar si a su alta demanda está contribuyendo una solicitud inadecuada por exceso.

La segunda manera indirecta para detectar la inadecuación en la solicitud de las pruebas de laboratorio es mediante estudios que muestren la diferencia de utilización de las pruebas en distintas áreas geográficas, y también comparar esta solicitud con lo recomendado en las guías clínicas y teniendo en cuenta la prevalencia de las distintas enfermedades en nuestro país. Un ejemplo de este tipo de estudios es los realizados por REDCONLAB, una red de intercambio de conocimiento entre los laboratorios, que comparan la demanda en la solicitud de pruebas desde AP y desde el servicio de urgencias hospitalario (SUH).

Como ejemplo, en la primera edición de los estudios REDCONLAB, con solo ocho participantes de la Comunidad Valenciana [30], se observó que, la solicitud de calcio sérico en nuestro departamento de salud estaba muy por debajo de la del resto de los departamentos de salud participantes. Además de esta diferencia en la solicitud de calcio sérico y como refirió la editorial del mismo ejemplar de la revista [31], la solicitud de ácido úrico en suero desde AP parecía ser inadecuada por exceso en los ocho laboratorios participantes. El argumento emitido en la editorial para realizar esta afirmación fue que, tanto en Suecia como en España la indicación para la solicitud de ácido úrico es la sospecha de gota en paciente sintomático; puesto que la prevalencia de gota es similar en ambos países, la demanda en los laboratorios españoles parecía muy elevada al compararla con la solicitud sueca. También se refirió en dicha editorial un potencial exceso de solicitud de hierro en los ocho departamentos participantes, pues en cada uno era más elevada que la de transferrina en suero, lo que implica solicitud de hierro sérico aislada.

En la tercera y cuarta ediciones de REDCONLAB, ya con 76 y 110 participantes, respectivamente, que cubría, en la cuarta edición, el 60 % de la población española, se observó una solicitud por defecto de HbA_1c_ [32, 33] y de albúmina en orina [34] ya que la solicitud era insuficiente para el diagnóstico de la diabetes y del daño renal, respectivamente, y la monitorización del paciente con hipertensión y diabetes, acorde a las guías y a la prevalencia de ambas enfermedades en España.

Sin embargo, se observó una solicitud en exceso de marcadores de anemia, como ferritina, transferrina, vitamina B12 y ácido fólico [35], y también de marcadores de función tiroidea [36].

En general, las 10 pruebas que generan más gasto [21] en muchos de los laboratorios españoles coinciden, en muchos casos, con lo hallado con exceso de solicitud en los estudios de REDCONLAB, como calcidiol [37], ferritina [35], tirotropina [36], proteína C reactiva [38], procalcitonina [39] o marcadores de función hepática [40, 41].

El resultado final de estos estudios de “benchmarking” o comparación de la solicitud de pruebas de laboratorio fue una mejora en el uso de estas, con lo que se demuestra la utilidad de estos estudios para identificar la inadecuación en la solicitud de pruebas, e incluso para su corrección [42]. La experiencia de los autores indica que, el conocimiento de la inadecuación estimula la implantación de IGD. Por ejemplo, la detección del defecto en la solicitud de calcio sérico en el departamento de salud llevó a la implantación de la IGD para mejorar el diagnóstico del hiperparatiroidismo primario [43], que se expondrá más adelante en este artículo.

### Selección de la prueba y el entorno donde establecer la IGD

La segunda etapa en el proceso de adecuación de la demanda es la selección de la prueba de la que corregir el exceso o el defecto de solicitud, y el entorno donde establecer la IGD. Esta etapa es clave para conseguir el éxito en la IGD.

**Figura 3:**
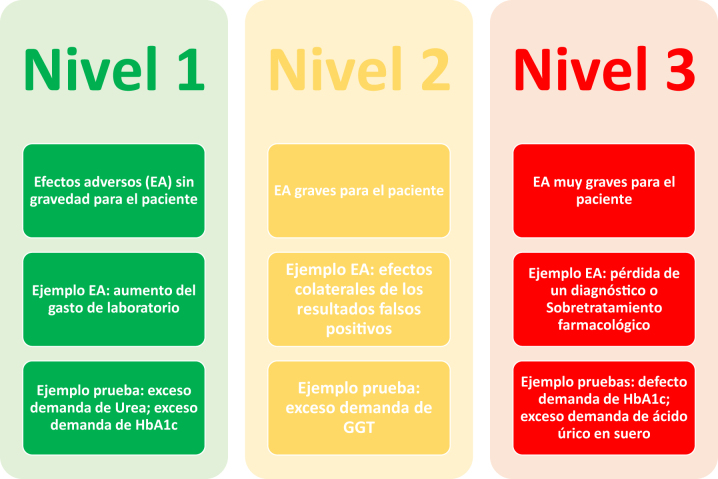
Nivel de las pruebas en función del efecto adverso que causa la inadecuación. EA: efectos adversos; HbA_1c_: hemoglobina glicosilada.

Las pruebas detectadas como inadecuadamente solicitadas se pueden clasificar en función del efecto adverso que genera su inadecuación en la demanda, en riesgo bajo, medio o alto (Figura 3). Se pretende evaluar el riesgo, las consecuencias o los efectos adversos del exceso o del defecto en la solicitud de la prueba, además de las consecuencias financieras [44]. Una prueba que su defecto en la demanda provocara una pérdida de diagnóstico precoz, lo que evitaría consecuencias graves (por ejemplo, la vitamina B12 para el déficit), se considerará de riesgo alto. Una prueba que no tuviera un precio elevado y su exceso de demanda solo implicara más gasto en reactivo sin tener muchas repercusiones sobre la seguridad del paciente se consideraría de bajo riesgo.

Una vez clasificadas las pruebas, se trata de corregir la demanda de aquellas pruebas que se han clasificado como de alto riesgo [44].

Respecto al entorno, o la población diana sobre la que instaurar la IGD, se sigue el principio de Pareto, implantando las intervenciones principalmente en AP o en el SUH, ya que, mediante poco esfuerzo, el diseño de una única IGD, impactará positivamente en muchos pacientes.

### Generación de la idea, prediseño y diseño de la IGD

Las siguientes tres fases consecutivas son clave en el proceso de corregir la demanda. En estas fases – generación de la idea, prediseño y diseño – hay dos puntos que son de especial importancia.

El primer punto es que las IGD sean automáticas, basadas en los sistemas de información. Este tipo de intervenciones son las únicas que sistemáticamente se mantienen en el tiempo. Las estrategias de tipo educacional funcionan al principio, pero casi irremediablemente “caen en el olvido”, incluso compartiendo a tiempo real con los clínicos la monitorización de su mejora. Como ejemplo, se muestra el caso de la corrección del exceso de demanda de proteína C reactiva en nuestro hospital; tras un protocolo consensuado con diferentes especialistas del hospital, la demanda mejoró, pero indefectiblemente empeoró posteriormente [45].

### El segundo punto clave en estas fases es la comunicación y el consenso con el clínico solicitante [17]. Establecimiento de la IGD

La siguiente etapa es el establecimiento de la IGD. Esta fase debe tener una duración concreta, para poder analizar su funcionamiento y decidir si continuar o no con la IGD.

### Monitorización de la IGD – indicadores de proceso e indicadores de resultado

Por último, pero no menos importante, y para siempre mientras la IGD esté en marcha habrá que monitorizar, también de manera automática la intervención. Esta monitorización se hará mediante indicadores de proceso (aumento o descenso de la demanda) y también, y lo más importante, mediante indicadores de resultado. Estos indicadores de resultado proporcionan información acerca de la mejora que ha supuesto la IGD para el paciente, para el ciudadano y para la sociedad. Estos indicadores de resultado pueden ser, por ejemplo, el número de casos detectados en un periodo de tiempo, los años de vida ganados respecto al número de casos detectados, el coste por caso detectado o número de tratamientos innecesarios por caso detectado. Al final, lo que se pretende medir es el impacto de la IGD en el paciente, en el ciudadano y en la sociedad. Por ejemplo, un indicador de resultado que se utiliza para monitorizar una IGD es el número de casos de pancreatitis aguda diagnosticados en el SUH mediante la IGD. Otro ejemplo interesante, es la disminución de la prescripción inadecuada de alopurinol cuando se adecúa la solicitud de ácido úrico en suero.

## IGD para corregir el uso excesivo de pruebas de laboratorio – adecuación de la demanda en exceso

**Figura 4:**
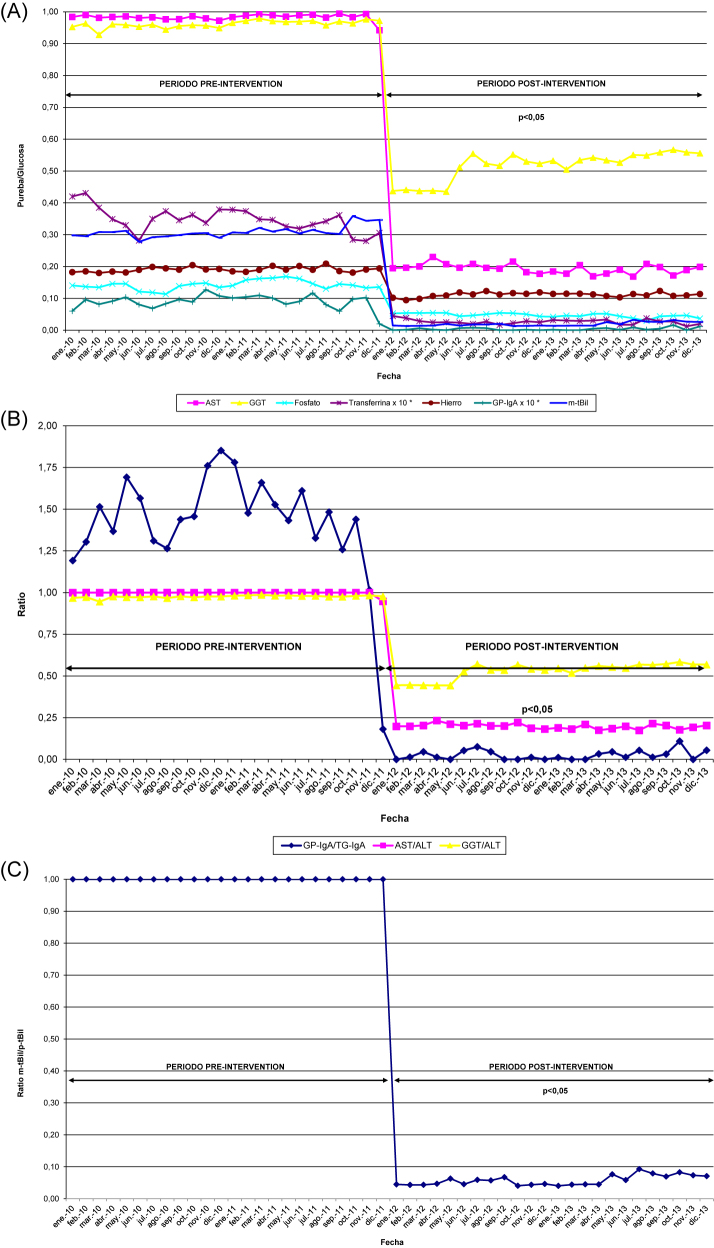
Indicadores de proceso. (A) Número de pruebas/número de glucosas. (B) Ratio de pruebas relacionadas. (C) m-tBil/p-tBil. *El valor del indicador transferrina/glucosa y GP-IgA/glucosa se multiplica por diez, para evitar el efecto escala. AST, aspartato aminotransferasa; ALT, alanina aminotransferasa; GGT, gamma-glutamil transpeptidasa; GP-IgA, anticuerpos frente a la gliadina deaminada de tipo IgA; m-tBil, bilirrubina total medida; p-tBil, bilirrubina total pedida; TG-IgA, anticuerpos frente a transglutaminasa tisular de tipo IgA.

Como ejemplo de que las IGD siempre deben ser automáticas, basadas en los sistemas de información y que deben ser monitorizadas en el tiempo mediante indicadores de proceso, se muestran las primeras intervenciones que se establecieron en nuestro departamento de salud [46], en qué se basó su diseño (Tabla 1) y como se observó la disminución en la medida de todas las pruebas (Figura 4), y en este caso como indicador de resultado, el ahorro en costes de reactivo.

**Tabla 1:** Principales Intervenciones de Gestión de la Demanda (IGD) en exceso.

	Prueba eliminada	Perfil	Acción
**1er grupo de estrategias**			

Eliminar pruebas de perfiles	AST	Perfil básico de salud (ALT, AST, hemograma, colesterol, creatinina, GGT, glucosa, triglicéridos), hígado (ALT, AST, GGT, tBil)	AST se añade de nuevo y se procesa si la ALT presenta valores patológicos
	GGT	Perfil básico de salud	–
	Fosfato	Perfil reumatología (proteína C-reactiva, calcio, creatinina, hemograma, VSG, glucosa, fosfato, factor reumatoide)	Fosfato se añade de nuevo y se procesa si el calcio presenta valores patológicos

	Prueba eliminada	Condición para eliminar	Acción
**2º grupo de estrategias**			

Eliminar pruebas de peticiones	Transferrina	Si se solicita simultáneamente con ferritina	Transferrina se añade de nuevo y se procesa si ferritina >400 ng/mL
	Hierro	Si no está solicitado simultáneamente con ferritina o transferrina	–
	25-OH-Vitamina D	Si la solicitud no está asociada a un diagnóstico relacionado	–

	Prueba cambiada	Condición para cambiar la prueba	Acción
**3er grupo de estrategias**			

Cambiar pruebas	Anticuerpos frente a la gliadina deaminada IgA	Paciente mayor de 2 años de edad	Se añaden los anticuerpos frente a la transglutaminasa tisular de tipo IgA

	Prueba no medida	Condición para evitar la medición	Acción
**4º grupo de estrategias**			

No medir la prueba	Bilirrubina total	Si el valor del índice ictérico está por debajo de 34,2 mmol/L (2 mg/dL)	Se añade un comentario como resultado automático

Todas las pruebas hacen referencia a su determinación sérica, excepto VSG que su determinación se realiza en sangre total. ALT, alanina aminotransferasa; AST, aspartato aminotransferasa; GGT, gammaglutamiltranspeptidasa; tBil, bilirrubina total; VSG, velocidad de sedimentación globular.

Las IGD basadas en intervalo mínimo de repetición (IMR) son muy fáciles de implementar. Solo se necesita disponer del SIL, con su base de datos de pacientes. Y existe mucha bibliografía al respecto, estando muchas basadas en los IMR de pruebas en ML de Reino Unido publicadas por el Royal College of Pathologists [47].

Los IMR se definen como el tiempo mínimo que ha de transcurrir antes de repetir una prueba, basándose en las propiedades de la prueba y la situación clínica en la que se utiliza la misma. También hay que tener en cuenta que existen pruebas que solo deben ser medidas una vez en la vida, como son los estudios genéticos o algunos anticuerpos cuando el resultado de estos es positivo. Puede incluir a todos los pacientes atendidos, o un entorno especifico, como AP, hospitalización, una combinación de ellos, etc. Se basa en que, si la prueba se ha solicitado en un periodo previo de días o de meses determinados, no se mide y se explica mediante un comentario que incluye el resultado previo.

La Tabla 2 muestra los IMR que están establecidos en nuestro laboratorio, incluyendo la prueba afectada, el tiempo, el entorno en que se aplica y la referencia bibliográfica.

**Tabla 2:** Intervalos mínimos de repetición.

Parámetros analíticos	Alcance	Diseño intervención	Indicador de proceso	Indicador de resultado	Tipo gestión de resultado
Ag prostático especifico (PSA)	ING	Se anula la prueba si está solicitada en los tres días previos [28].	Número de pruebas no procesadas	Ahorro	Se informa mediante el resultado anterior con un comentario
	Todos	Se anula la prueba si está solicitada en los tres meses previos.			
Calcidiol (25 hidroxicolecalcifero)	Todos	No se realiza si la determinación está realizada en los seis meses previos y si el resultado previo no patológico.			
Cianocobalamina (vitamina B12)	Todos	No se realiza si la determinación está realizada en los tres meses previos y si el resultado previo no patológico [57].			
	ING	No se realiza si la determinación está realizada en los tres días previos [28].			
Colesterol HDL	ING	No se realiza si la determinación está realizada en los siete días previos [28].			
Factor reumatoide	Todos	No se realiza si la determinación está realizada en el año previo.			
	ING	No se realiza si la determinación está realizada en los tres días previos [28].			
Ferritina	Todos	No se realiza si la determinación está realizada en los tres meses previos y si el resultado previo no patológico.			
	ING	No se realiza si la determinación está realizada en los tres días previos [28].			
Folato	ING	No se realiza si la determinación está realizada en los tres días previos [28].			
Hierro	ING	No se realiza la determinación si ha sido solicitada en los tres días previos [28].			
Inmunoglobulinas (IGA, IGG, IGM)	ING	No se realiza la determinación si ha sido solicitada en los tres días previos [28].			
Transferrina	ING	No se realiza la determinación si ha sido solicitada en los tres días previos [28].			
Trigliceridos	ING	No se realiza la determinación si ha sido solicitada en los tres días previos [28].			
Anticuerpos Anti-nucleares (ANA)	Todos	No se realiza la determinación si ha sido solicitada en los tres meses previos, si el resultado previo no patológico			
Screening enas	Todos	No se realiza la determinación si ha sido solicitada en los tres meses previos.			
Heglobina glicosilada	ING	No se realiza la determinación si ha sido solicitada en los siete días previos [28].			
	AP	No se realiza la determinación si ha sido solicitada en los dos meses previos.			

AP, atención primaria; ING, ingresados.

como puede observarse en la Tabla 2, en las establecidas en pacientes durante su ingreso, el IMR es de días. Dependiendo de la prueba, está establecido de 3 a 7 días como IMR [30], siendo de meses en los demás entornos. Aquellos que carecen de referencia es porque se han establecido por los autores en base a la experiencia propia. Como se ha expuesto con anterioridad, una vez detectada en qué pruebas hay exceso en solicitud, es clave cuáles elegir. En nuestro departamento se ha clasificado de alto riesgo para el paciente el exceso de solicitud de ácido úrico en suero, gamma-glutamil transpeptidasa (GGT) y marcadores tumorales desde AP y para estas pruebas, se ha diseñado IGD para corregir la inadecuación de la solicitud por exceso [48], [49], [50].

En el caso del ácido úrico, con el exceso de solicitud se corre el riesgo de tratar a pacientes con hiperuricemia asintomática, de hecho, como indicador de resultado se utiliza la prescripción de alopurinol en AP y esta disminuyó significativamente tras adecuar la solicitud de ácido úrico al laboratorio, corrigiendo su exceso de demanda [48]. Cuando el exceso de solicitud es de GGT o marcadores tumorales, el riesgo es generar un elevado número de resultados falsos positivos, con sus colaterales efectos adversos (aumento del número de consultas, aumento del número de pruebas diagnósticas innecesarias, yatrogenia, etc.). Además, es la fosfatasa alcalina, y no la GGT, la prueba de función hepática de segundo nivel tras la alanina aminotransferasa (ALT), para valorar la colestasis, y los marcadores tumorales no deben ser utilizados como screening en AP, y menos aun solicitando simultáneamente varios de ellos, base en que se estableció nuestra IGD.

No queremos dejar de incluir en esta revisión y en este apartado de adecuación de la demanda en exceso algunas intervenciones que, aunque no corrigen el exceso de solicitud, sí que disminuyen los costes al informar algunas pruebas mediante otras a bajo coste. Este tipo de intervenciones son fáciles de instaurar, pues para su diseño, no se necesita más que el SIL. Ejemplos de este tipo de intervenciones serían informar la bilirrubina basándonos en el resultado del índice ictérico [51], y la albúmina en orina, mediante los resultados semicuantitativos de la tira de urianálisis de creatinina y albúmina [52].

Además de la AP, el SUH ha sido participe de una atención estrecha por el laboratorio a lo largo de estos últimos años [53]. En este entorno, se reemplazó el procesamiento de proteínas totales plasmáticas por la albúmina, se informa de forma automática el calcio corregido por albúmina cuando existe hiper o hipocalcemia, se utiliza la lipasa en plasma como primer marcador para el diagnóstico de pancreatitis y dependiendo del resultado se realiza o no la amilasa [54], y siempre de forma automática se mide magnesio en plasma cuando el paciente presenta hipocalcemia, hipopotasemia o valores altos de lipasa [55].

Por último, y respecto a las IGD para corregir el exceso de demanda, hay que tener especial cuidado de SOLO establecer las adecuadas para corregir dicho exceso. Y NUNCA dejar de realizar pruebas clave y útiles para la prevención, diagnóstico, monitorización y tratamiento de la enfermedad. Ejemplos claros serían no dejar nunca de medir ferritina o vitamina B12 porque el resultado de la hemoglobina sea normal, ya que la primera prueba es clave en el diagnóstico de déficit de hierro, y la segunda es clave para el diagnóstico de déficit de vitamina B12, ocurriendo ambos déficits, en muchos pacientes, sin una concomitante anemia. Hay que recordar que la demencia en un paciente con déficit de vitamina B12 puede ser irreversible, si no se trata en los primeros seis meses [56].

## IGD para corregir la infrautilización de pruebas de laboratorio – adecuación del defecto de demanda

El efecto adverso de la inadecuación de la solicitud por defecto es, como se apuntó anteriormente, la no prevención, las pérdidas de diagnósticos, la no monitorización o el tratamiento inadecuado, es decir, no se alcanza la misión del laboratorio.

Antes de enumerar las IGD para corregir el defecto en la solicitud de pruebas de laboratorio, solo apuntar que en la mayoría de ellas existe de forma conjunta un exceso y un defecto de demanda. Esto ocurre, por ejemplo, con la vitamina B12, de la que existe un exceso de solicitud que se ha corregido mediante una IGD basada en el IMR [57], y un defecto que se corrige mediante una IGD que consiste en registrar la prueba de forma automática en pacientes de AP cuando el volumen corpuscular medio (VCM) de los hematíes es superior a 100 fL [58, 59] y con otra IGD en pacientes en tratamiento crónico con inhibidores de la bomba de protones a los que no se les ha solicitado la prueba [58].

Otro ejemplo de esta dualidad entre el exceso y el defecto en la solicitud de pruebas se encuentra en la ferritina. En esta prueba también existe un exceso en la solicitud que se corrige mediante una IGD basada en IMR [60], y un defecto, que se corrige, y así se detectan pacientes con déficit de hierro, mediante el registro automático de la prueba en pacientes de AP con diagnóstico de alopecia [61], o en pacientes menores de 14 años aunque no presenten anemia [62]. En ambos ejemplos, la IGD para corregir el defecto en la demanda está supeditada a que la prueba, vitamina B12 o ferritina, respectivamente, no se hubiera solicitado ni medido en el año previo. Otro ejemplo de esta convivencia entre el exceso y el defecto en la solicitud lo constituirían las determinaciones de IgE específica desde AP, que se encuentran solicitadas en muchos casos por exceso, pero también existe defecto en su solicitud, y por tanto, estas pruebas también precisan del establecimiento de IGD [63].

En el nuevo modelo de laboratorio, el Laboratorio Líder [18], las estrategias no solo se centran en corregir el exceso en la demanda, sino que también se corrige el defecto de la demanda.

Una de las determinaciones de las que se corrige el defecto de la demanda es el calcio sérico. En pacientes de AP, cuando esta determinación no ha sido medida en los últimos tres años y el paciente es mayor de 45 años, automáticamente se registra y se determina. En el SUH, cuando se atiende a un paciente con historia previa de cáncer se realiza la determinación de calcio sérico de forma automática. Con estas dos estrategias, se diagnostican pacientes con hiperparatiroidismo primario (HPTP) [43] o con hipercalcemia maligna [64], respectivamente. En esta última IGD para el diagnóstico de hipercalcemia maligna desde el SUH, se accede a tiempo real a los datos de la historia clínica del paciente mediante un CDS. En ambas estrategias, el indicador de resultado para la monitorización de las mismas es el número de casos detectado, de HPTP y de hipercalcemia maligna, respectivamente.

Otro ejemplo de IGD para corregir el defecto en una solicitud, es el registro y la determinación automática de la HbA_1c_, cada 3 años en pacientes de AP mayores de 45, y niveles de glucosa en la misma petición superiores a 110 mg/dL [65]. También se determina la HbA_1c_ en pacientes entre 25 y 46 años y valores patológicos de triglicéridos y/o HDL colesterol cuando la HbA_1c_ no se ha solicitado en el año previo o cuando la glucosa en esa misma petición es >100 mg/dL [20]. El indicador para monitorizar las estrategias es el número de pacientes identificados con diabetes.

También se realiza adecuación de la solicitud de vitamina B12 [64], contabilizándose como indicador de resultado el número de nuevos pacientes identificados con déficit grave de vitamina B12.

Otra prueba que también se adecua es el magnesio. En el SUH, el magnesio sérico se registra automáticamente en pacientes con hipocalcemia y/o hipopotasemia [52, 66] y en AP, además de en pacientes que presenten estas alteraciones electrolíticas, también se añade en pacientes con diabetes y pacientes mayores de 65 años siempre que no lo tengan solicitado en el año previo [67]. Para estas intervenciones, el indicador de resultado para su monitorización es el número de pacientes identificados con hipomagnesemia.

Como se puede observar, desde el laboratorio se pueden establecer IGD para detectar enfermedad oculta. Muy interesante puede resultar la detección de anticuerpos anti-células parietales en pacientes con un déficit grave de vitamina B12 [68] prueba que, tras ser positiva, nunca más tiene que volver a medirse.

Otra estrategia que permite realizar un cribado de enfermedad oculta es la identificación de pacientes con mieloma. El SIL registra automáticamente las inmunoglobulinas séricas cuando las proteínas totales en suero son >80 g/L por primera vez en pacientes de AP. Si una de las inmunoglobulinas presenta un valor por encima y otra por debajo de sus rangos de referencia, el SIL registra automáticamente una electroforesis de proteínas séricas (proteinograma) [69].

Es clave, puesto que forma parte de la misión del laboratorio, adecuar la solicitud de pruebas con el fin de mejorar la monitorización de las enfermedades, siempre acorde a las guías clínicas. Desde el laboratorio, y como siempre de forma consensuada con el médico, es posible adecuar la solicitud de HbA_1c_, las pruebas del perfil lipídico y la albúmina en orina en el paciente diabético, registrando estas pruebas de acuerdo a la cadencia indicada en las guías de seguimiento de la diabetes [20]. También es posible adecuar la solicitud de albúmina en orina en el paciente hipertenso siguiendo las recomendaciones de las guías clínicas [70]. Con estas dos IGD se mejora la adherencia a las guías clínicas y con ello el pronóstico de unas enfermedades crónicas de muy alta prevalencia, tal como se observa con la disminución de los niveles de HbA_1c_ en los pacientes diabéticos de nuestro departamento de salud [18].

Aunque *a priori* estas IGD para adecuar la infrautilización de pruebas de laboratorio resulten en un aumento del gasto en reactivo de aquella prueba que se añade automáticamente; en realidad son estas IGD las que suponen un mayor ahorro al laboratorio clínico y al sistema sanitario. El diagnóstico precoz y la monitorización adecuada mejora considerablemente el pronóstico de la enfermedad. Y tal como se ha mostrado estas intervenciones se pueden aplicar en enfermedades muy prevalentes, por lo que también el beneficio a la sociedad es altísimo.

## Conclusiones

Desde el laboratorio, se destaca la importancia crítica del diseño e implementación de IGD, preferiblemente automáticas y fundamentadas en sistemas de información. La colaboración estrecha con los clínicos es esencial para garantizar un flujo eficiente de información. La monitorización continua a lo largo del tiempo mediante indicadores de proceso es esencial, ya que estos reflejan los ajustes realizados en la solicitud de pruebas, mostrando cómo el procesamiento se ve afectado por correcciones de excesos o defectos.

Adicionalmente, la incorporación de indicadores de resultado permite visualizar cómo estas IGD contribuyen a mejorar los resultados para el paciente. En este innovador modelo de Laboratorio Líder, que no solo participa, sino en muchas ocasiones toma decisiones clínicas, su misión trasciende la mera medición de pruebas de laboratorio. La prevención, diagnóstico, monitorización y tratamiento de enfermedades se vuelven aspectos fundamentales [71].

En este contexto, la figura del profesional de laboratorio emerge como clave, desempeñando un papel fundamental en la gestión efectiva de estos sistemas y contribuyendo al éxito integral del enfoque centrado en el paciente.
